# Surgical Repair of Ventricular Septal Defect; Contemporary Results and Risk Factors for a Complicated Course

**DOI:** 10.1007/s00246-016-1508-2

**Published:** 2016-11-21

**Authors:** Maartje Schipper, Martijn G. Slieker, Paul H. Schoof, Johannes M. P. J. Breur

**Affiliations:** 1 Department of Pediatric Cardiology, Wilhelmina Children’s Hospital, University Medical Center Utrecht, PO Box 85090, 3508 AB Utrecht, The Netherlands; 2 Department of Pediatric Cardiothoracic Surgery, Wilhelmina Children’s Hospital, University Medical Center Utrecht, PO Box 85090, 3508 AB Utrecht, The Netherlands

**Keywords:** Ventricular septal defect, Congenital heart disease, Cardiac surgery, Risk factors, Pediatric cardiology, Pediatric cardiac surgery

## Abstract

Surgical closure of the ventricular septal defect is the most commonly performed procedure in pediatric cardiac surgery. There are conflicting data on weight at operation as risk factor for a complicated course. We performed a retrospective evaluation of mortality and morbidity in all patients undergoing surgical ventricular septal defect closure at our institution between 2004 and 2012 to identify risk factor for a complicated course. Multivariate logistic regression modeling was performed to identify risk factors for a complicated course. 243 patients who underwent surgical ventricular septal defect closure were included. Median age at operation was 168.0 days (range 17–6898), the median weight 6.0 kg (range 2.1–102.0). No deaths occurred. Two patients (0.8%) required a pacemaker for permanent heart block. Five patients (2.1%) underwent reoperation for a hemodynamically important residual ventricular septal defect. No other major adverse events occurred. No risk factors for major adverse events could be established. Multivariate analysis identified a genetic syndrome, long bypass time and low weight at operation as independent risk factors for a prolonged intensive care stay (>1 day) and prolonged ventilation time (>6 h). Contemporary results of surgical VSD closure are excellent with no mortality and low morbidity in this series. Although it is associated with increased ventilation time and a longer hospital stay, low bodyweight at operation is not associated with an increased risk of complications or major adverse events in our series.

## Introduction

With an incidence of 2–5%, the ventricular septal defect (VSD) is the most common congenital malformation of the heart [1, 2]. Therefore, VSD repair is the most commonly performed pediatric cardiac operation. Recent reports have indicated a very low incidence of postoperative complications [3]. Nevertheless complications still occur and it is important to identify associated risk factors. With the introduction of device closure of VSD, contemporary results of surgical repair are necessary to provide current benchmarks of treatment.

Recently Anderson et al. [4] found that young age (<6 months) and low bodyweight were risk factors for complications. This, however, contrasts with data published by Kogon et al. [5]. Therefore, risk factors for a complicated course after VSD closure vary between centers.

The purpose of this study is to evaluate the results of surgical VSD closure in a recent single center cohort and to define risk factors for a complicated course.

## Patients and Methods

### Patients

All patients who underwent surgical closure of a simple VSD between March 1, 2004 and December 31, 2012 at the Children’s Hospital, University Medical Center Utrecht, The Netherlands, were retrospectively reviewed. A simple VSD was defined as an isolated VSD or a VSD with concomitant atrial septal defect/ patent foramen ovale, patent ductus arteriosus or mildly stenotic/regurgitant semilunar valves. VSD closure was the main indication for surgical intervention in all patients. Patients with prior pulmonary artery banding were also included. Patients with more complex cardiac anomalies, including atrioventricular septal defect, coarctation of the aorta, severe stenosis or insufficiency of the semilunar valves and tetralogy of Fallot, were excluded. Medical records of these patients were retrospectively reviewed with permission from the institutional review board of the University Medical Center Utrecht, The Netherlands.

Two hundred forty-three patients met the inclusion criteria. We collected preoperative, perioperative and postoperative data from the echocardiography reports, perfusion reports, clinic, inpatient and operative notes for all the patients. Outcomes were assessed through the latest postoperative visit before inclusion. All patients that met the inclusion criteria were analyzed.

The preoperative characteristics, including sex, gestational age, birth weight, cardiac and non-cardiac comorbidity, age at operation, bodyweight at operation, days in hospital preoperatively, days ventilated preoperatively, type of VSD, indication for surgery, product of multiple gestation and underlying genetic condition, were examined. Follow-up duration was calculated in years using the date of operation and the date of the start of the database. VSD types were subdivided in four groups, perimembranous, muscular, doubly committed and multiple based on transthoracic echocardiography. The indications for operation were organized in three groups: volume load (failure to thrive or congestive heart failure), obstruction (right ventricular outflow tract obstruction, aortic insufficiency or double-chamber right ventricle) and pulmonary (elevated pulmonary vascular resistance). This classification was based on the information provided by the referring cardiologists.

The surgical technique used, surgeon who operated, cross-clamp and bypass times and the need for a second bypass run were assessed as operative factors. Surgical techniques were primary closure and patch closure.

Surgical outcome and complications were assessed according to international standards [6] and included length of stay, length of postoperative ventilation, residual VSD (defined as flow across the ventricular septum by echocardiography directly after operation or at first outpatient clinic visit), incidence of reoperation for a significant residual VSD, reintubation, refixation of the sternum, wound infection, post-pericardiotomy syndrome, chylothorax, transient or complete heart block, seizure, rehospitalization, circulatory arrest, stroke, renal failure, neurological deficit, paralyzed diaphragm, need for postoperative mechanical circulatory support and death. Unintended reoperation, heart block requiring a permanent pacemaker, circulatory arrest and death were considered major adverse events.

### Surgical Technique

Median sternotomy was the used surgical approach in all patients. All patients underwent cardiopulmonary bypass. Four surgeons from the Children’s Hospital, University Medical Center Utrecht, The Netherlands performed the surgeries (three surgeons performed 97.5% of the surgeries). The surgeon decided whether primary closure or closure with a patch was indicated based on their own expertise. During the same operation, the surgeon also performed atrial septal defect closure, patent foramen ovale closure, patent ductus arteriosus ligation, infundibular muscle resection, valvuloplasty and/or division when needed.

Before the operation, all patients underwent transthoracic echocardiography during outpatient clinic visits. All patients received an epicardial or a transesophageal echocardiographic re-evaluation just prior to surgery. An outcome echo was made right after VSD closure during ultrafiltration to assess the surgical result. Postoperatively all patients underwent another transthoracic echocardiography during hospital stay prior to discharge.

### Statistical Analysis

Statistical analyses were performed using IBM SPSS Statistics (version 20.0.0). The Kolmogorov-Smirnov test was used to verify whether our data were normally distributed or not. Only birth weight and the logarithm of aortic cross-clamp time and bypass time were normally distributed, all the other continuous variables were non-normally distributed. For the normally distributed data, we used means and standard deviations to describe the variables, for the not normally distributed data we used medians and ranges to describe the variables. Frequencies and percentages were used for categorical data. For analysis the unequally distributed variables were dichotomized. Univariate analysis was done using a univariate logistic regression model. Determinants that were univariately associated (*P* < 0.1) with the outcome were included in a multivariate logistic regression model to verify whether they were independent predictors.

## Results

### Patient Characteristics

A total of 243 patients underwent surgical VSD closure during the study period. Patient characteristics are presented in Table 1. Forty-nine percent of patients were male. Median age at operation was 168 days with a corresponding median bodyweight at operation of 6.0 kg. Fifty-three percent of the patients was 6 months or younger at the time of operation. An underlying genetic syndrome was present in 18.1%. Concomitant anomalies were found in multiple patients. Mean follow-up duration was 3.1 years.

**Table 1:** Patient characteristics (*n* = 243)

Sex	
Boy (%)	118 (48.6)
Girl (%)	125 (51.4)
Gestational age (weeks, median with min–max)^a^	39.0 (29–42)
Birth weight (g, mean with SD)^b^	3 141 (±778)
Weight at operation (g, median with min–max)	6 000 (2100–102,000)
Age at operation (days, median with min–max)	168 (17–6898)
Age groups	
≤ 6 months (%)	128 (52.7)
>6 months (%)	115 (47.3)
Genetic syndrome	
Trisomy 21 (%)	31 (12.8)
22q11 (%)	3 (1.2)
Other (%)	10 (4.1)
Concomitant cardiac defects	
Patent foramen ovale (%)	55 (22.6)
Atrial septal defect (%)	53 (21.8)
Patent ductus arteriosus (%)	30 (12.3)
Pulmonary valve stenosis (%)	18 (7.4)
Follow-up duration (years, median with min–max)	3.1 (0.3–9.1)

*SD* standard deviation

^a^ *n* = 227, from 16 patients gestational age was unknown

^b^ *n* = 228, from 15 patients birth weight was unknown

### Operative Characteristics

Table 2 lists the operative characteristics. 205 patients were outpatient before surgery. Two hundred thirty-three patients were not ventilator dependent preoperatively. Two patients had a postoperative intensive care unit (ICU) stay of more than 8 days. One patient had an ICU stay of 21 days due to failure to extubate because of laryngotracheobronchomalacia. Another patient had an ICU stay of 31 days because of a big muscular VSD which remained unnoticed during surgery of the target VSD which caused pulmonary hypertension, combined with pneumonia and upper respiratory tract infection. A total of 188 patients had a total postoperative stay of 1 week or less. In most cases an infection (mostly respiratory tract) was the cause of a prolonged stay. The majority of patients were extubated within 24 h (*n* = 210), 31 patients were extubated within 6 days. The two patients with a prolonged ICU stay were ventilated for 20 and 28 days, respectively. In 124 patients (51%) postoperative transesophageal or transthoracic echocardiography showed residual Doppler signals across the septum indicating residual VSD. The majority of defects were very small. Most of these residual shunts (71%) disappeared during a mean follow-up period of 3.1 years.

**Table 2:** Operative characteristics (*n* = 243)

Indication for operation	
Volume load (%)	168 (69.1)
Obstruction (%)	52 (21.4)
Pulmonary (%)	23 (9.5)
Ventricular septal defect type	
Perimembranous (%)	192 (79)
Muscular (%)	14 (5.8)
Doubly committed (%)	24 (9.9)
Multiple (%)	13 (5.3)
Surgical technique	
Primary closure (%)	47 (19.3)
Patch (%)	196 (80.7)
Hospitalization (days)	
Preoperative (median with min–max)	0.0 (0–78)
Postoperative ICU (median with min–max)	1.0 (1–31)
Postoperative total (median with min–max)	5.0 (3–65)
Mechanical ventilation (days)	
Preoperative (median with min–max)	0.0 (0–40)
Postoperative (median with min–max)	0.3 (0–28)
Bypass time (min, mean with SD)	63.5 (±22.5)
Aortic cross-clamp time (min, mean with SD)	41.2 (±18.1)

Indications for operation were volume load (failure to thrive or congestive heart failure), obstruction (right ventricular outflow tract obstruction, aortic insufficiency or double-chamber right ventricle) or pulmonary (elevated pulmonary vascular resistance)

*SD* standard deviation

### Complications

Table 3 summarizes the examined complications. Two percent of all patients needed reoperation because of a significant residual VSD. One patient had a seizure 1 day postoperatively. The cause of the seizure remained unclear. There was no permanent neurologic deficit afterward. The 30 day and in hospital mortality was 0%. Two patients died during late follow-up. One patient died 4 months after the operation during a readmission for pulmonary hypertensive crisis caused by previously undiagnosed severe pulmonary vein stenosis. The other patient died of severe dehydration 15 months after the surgery. These deaths were considered to be unrelated to VSD closure. In total 15.6% of the patients had one or more complications, whereas 2.9% of all patients had a major adverse event.

**Table 3:** Complications (*n* = 243)

Reintubation (%)	7 (2.9)
Reoperation for a significant residual VSD (%)	5 (2.1)
Refixation of the sternum (%)	6 (2.5)
Wound infection (%)	4 (1.6)
Postpericardiotomy syndrome (%)	4 (1.6)
Chylothorax (%)	1 (0.4)
Heart block	
Transient (%)	12 (4.9)
Permanent^a^ (%)	2 (0.8)
Seizure (%)	1 (0.4)
Rehospitalization (%)	6 (2.5)
Circulatory arrest (%)	0 (–)
Stroke (%)	0 (–)
Renal failure (%)	0 (–)
Neurological deficit (%)	0 (–)
Paralyzed diaphragm (%)	0 (–)
Need for postoperative mechanical circulatory support	0 (–)
Death (%)	0 (–)
Total complications^b^ (%)	38 (15.6)
Total major adverse event^c^ (%)	7 (2.9)

^a^Requiring pacing by a pacemaker

^b^Number of patients with one or more complications

^c^Accounted as major adverse events are reoperation, permanent heart block, circulatory arrest or death

### Predictors

A univariate analysis was performed to determine risk factors for a prolonged postoperative stay (ICU and total duration of stay), prolonged duration on mechanical ventilation, complications, major adverse events and postoperative infections other than wound infection (Table 4). A prolonged postoperative stay at the ICU was defined as a stay of more than one day. A prolonged postoperative stay in total was defined as a stay of more than 1 week. A prolonged duration on mechanical ventilation was defined as mechanical ventilation more than 6 h after surgery. Variables that were not normally distributed were dichotomized. Due to the rarity of complications and major adverse events multivariate analysis could only be performed for the duration of postoperative ICU stay, total length of stay and postoperative ventilation time (Table 5).

**Table 4:** Univariate analyses: riskfactors for a longer stay or complications

	Post-op time in total >1 week		Post-op time at ICU >1 day		Post-op ventilation time >6 h		Complications		Major adverse events		Post-op infection other than wound infection	
	OR (95% CI)	*P* value	OR (95% CI)	*P* value	OR (95% CI)	*P* value	OR (95% CI)	*P* value	OR (95% CI)	*P* value	OR (95% CI)	*P* value
Genetic syndrome	2.34 (1.16–4.75)	0.018	3.99 (2.00–7.95)	<0.001	3.27 (1.67–6.40)	0.001	1.25 (0.53–2.96)	0.608	0.75 (0.09–6.38)	0.791	2.74 (0.77–9.81)	0.121
Indication for operation^a^												
Obstruction	0	0.997	0.21 (0.09–0.49)	<0.001	0.20 (0.08–0.49)	<0.001	1.32 (0.57–3.06)	0.519	1.30 (0.25–6.93)	0.755	0.39 (0.05–3.21)	0.383
Pulmonary	1.09 (0.42–2.87)	0.853	2.50 (1.01–6.22)	0.049	2.35 (0.96–5.73)	0.061	2.23 (0.80–6.23)	0.128	0	0.998	1.91 (0.38–9.57)	0.434
Weight at operation <6000 g	10.11 (4.34–23.52)	<0.001	5.59 (3.14–9.96)	<0.001	5.82 (3.21–10.56)	<0.001	2.06 (1.00–4.25)	0.051	2.46 (0.47–12.92)	0.288	4.58 (0.97–21.65)	0.055
Longer aortic cross-clamp time	5.88 (2.57–13.47)	<0.001	4.62 (2.24–9.53)	<0.001	3.98 (1.94–8.14)	<0.001	1.45 (0.62–3.42)	0.395	2.02 (0.33–12.50)	0.451	1.42 (0.32–6.29)	0.643
Longer bypass time	10.86 (3.85–30.61)	<0.001	5.12 (2.15–12.22)	<0.001	4.69 (1.96–11.18)	<0.001	2.36 (0.82–6.78)	0.110	4.51 (0.51–39.53)	0.174	1.28 (0.20–8.12)	0.794
Secondary bypass run	1.75 (0.42–7.24)	0.440	1.28 (0.34–4.89)	0.718	0.89 (0.23–3.66)	0.875	0.67 (0.08–5.48)	0.705	0	0.999	0	0.999
Post-op infection other than wound infection	4.48 (1.31–15.30)	0.017	2.92 (0.83–10.25)	0.095	2.24 (0.66–7.56)	0.195	1.21 (0.25–5.83)	0.812	3.77 (0.41–34.33)	0.239	–	–
Date of operation before 01-01-2009	1.70 (0.93–3.12)	0.086	2.27 (1.33–3.86)	0.003	1.81 (1.06–3.10)	0.029	0.78 (0.38–1.61)	0.502	0.62 (0.12–3.24)	0.566	0.15 (0.02–1.17)	0.070

Complications are reintubation, reoperation for a significant rest VSD, refixation of the sternum, wound infection, postpericardiotomy syndrome, chylothorax, transient and permanent heart block, seizure, rehospitalization, circulatory arrest, stroke and death. Major adverse events are reoperation, permanent heart block, circulatory arrest, stroke, renal failure, neurological deficit, paralyzed diaphragm, need for postoperative mechanical circulatory support and death. Indications for operation were volume load (failure to thrive or congestive heart failure), obstruction (right ventricular outflow tract obstruction, aortic insufficiency or double-chamber right ventricle) or pulmonary (elevated pulmonary vascular resistance)

*OR* odds ratio, *95% CI* 95% confidence interval

^a^Reference group is the indication for operation volume load

**Table 5:** Multivariate analyses: riskfactors for a longer stay or ventilation time

	Post-op time in total >1 week		Post-op time at ICU >1 day		Post-op ventilation time >6 h	
	OR (95% CI)	*P* value	OR (95% CI)	*P* value	OR (95% CI)	*P* value
Genetic syndrome	2.07 (0.89–4.83)	0.092	3.13 (1.45–6.79)	0.004	2.45 (1.15–5.23)	0.020
Indication for operation^a^						
Obstruction	0	0.997	0.48 (0.18–1.27)	0.138	0.47 (0.17–1.32)	0.151
Pulmonary	0.60 (0.20–1.76)	0.347	1.35 (0.50–3.68)	0.558	1.35 (0.51–3.59)	0.548
Weight at operation <6000 g	5.28 (2.12–13.17)	<0.001	3.37 (1.72–6.60)	<0.001	3.57 (1.80–7.09)	<0.001
Longer bypass time	12.05 (3.51–41.33)	<0.001	4.52 (1.67–12.20)	0.003	3.94 (1.47–10.59)	0.006

Indications for operation were volume load (failure to thrive or congestive heart failure), obstruction (right ventricular outflow tract obstruction, aortic insufficiency or double-chamber right ventricle) or pulmonary (elevated pulmonary vascular resistance)

*OR* odds ratio, *95% CI* 95% confidence interval

^a^Reference group is the indication for operation volume load

A longer postoperative stay in total was predicted by the presence of a genetic syndrome, a weight of <6.0 kg, a longer aortic cross-clamp or bypass time and a postoperative infection other than wound infection. When analyzed multivariately weight at operation and a longer bypass time were still predictors for a longer postoperative hospital stay.

Patients with a genetic syndrome, with a weight of <6.0 kg at operation, with a longer bypass or cross-clamp time during operation and patients operated on before 01-01-2009 were more likely to have a longer stay at the ICU and a longer ventilation time. In the multivariate analysis a genetic syndrome, weight of <6.0 kg at operation and a longer bypass time again were identified as predictors for a longer postoperative stay at the ICU and a longer ventilation time.

In the univariate analysis no predictors were found for complications, major adverse events and postoperative infections.

## Discussion

### Contemporary Results

This study shows that the contemporary results of surgical VSD closure are excellent. There was no mortality and major adverse events occurred only in 2.9% of patients. These results are comparable with results of other recent studies [3–5, 7]. Currently, data on mortality for congenital heart surgery in Europe are collected in the European Association for Cardio-Thoracic Surgery (EACTS) Congenital Database. This database shows a 30 day mortality of 1.38% for patch repair and 1.84% for primary closure with a hospital mortality of, respectively, 1.57 and 2.17% [8]. Major adverse events are not yet registered in this database. Surgical VSD closure is a very safe procedure, and future studies should aim to further reduce complications and serious adverse events.

### Complications

Other studies show comparable results regarding the number of complications with permanent heart block occurring in 0.0–2.1% (0.85% this study) and reoperation in 0.0–4.9% (2.1% this study) [3–5, 7].

We found a high percentage of initial residual VSD’s of 51.0% in the present study. However, spontaneous closure occurred in 71.0% of these residual VSD’s during a limited follow-up period (mean of 3.1 years). Therefore, the eventual rate of spontaneous closure will probably even be higher. Reoperation for a hemodynamically important residual VSD, however, is extremely rare (2.1%). Other than endocarditis prophylaxis in case of patch closure, a hemodynamically insignificant residual VSD has no clinical consequences. Other studies reported residual VSD’s in different ways, with varying follow-up duration. One study found a residual VSD in 16% of the patients after a mean follow-up of 1.1 years, without reporting the initial count of residual VSD’s [3], whilst another study found residual VSD’s in 8% of the patients after a follow-up duration of 12 years [9], reporting the same count after a follow-up duration of more than 20 years [10]. These data suggest that spontaneous closure of residual VSD’s occurs relatively early after surgery. Our high rate of residual VSD’s may also be the result of the routine performance of transesophageal echocardiography in all patients that undergo cardiac surgery regardless of age. The transesophageal echocardiography microprobe that is used in small infants is not yet widely used. It allows us to detect even small residual VSD’s in the operating theater that may have spontaneously closed at the first postoperative transthoracic echocardiography.

### Risk Factors for Complications

A recent study showed that low bodyweight remains a significant predictor of morbidity [4]. This could not be confirmed by the present study. Our study had a low complication rate; therefore, only univariate analyses could be performed which showed that there was no significant risk factor for major adverse events or complications in general, including lower bodyweight or age. Our results suggest that surgical VSD closure should not be postponed for weight gain of the low bodyweight patients. However, the odds ratio for a weight <6.0 kg at operation for the risk at complications was 2.06 (95% CI 1.00–4.25) with a *P* value of 0.051; therefore, future research is needed to support this conclusion.

The current study shows that low bodyweight at operation is associated with a longer postoperative stay at the ICU and a longer postoperative ventilation time. This is in accordance with two other studies [4, 7], but denied by one other study stating that bodyweight does not influence these aspects [5].

In summary low bodyweight will prolong hospital stay but is not associated with complications or major adverse events. Therefore, surgical VSD closure is considered a safe procedure in all patients regardless of their bodyweight.

Patients with a genetic defect are also at risk for a longer postoperative stay at the ICU and a longer postoperative ventilation time. This may well be explained by complicating anomalies associated with the genetic defect as reported in previous studies [4].

### Future Perspectives

Only one study group described the long-term effects of surgical repair of a VSD at young age [10, 11]. According to the first study [10], patients may be at higher risk for a sick-sinus syndrome as 4% developed this after a mean follow-up of fifteen years. Aortic regurgitation was another late complication that was described in 16% of all patients in this study. This study described 22–34 years of follow-up from operations that took place between 1968 and 1980. The second study of the same cohort [11] with a follow-up up to 40 years shows systolic dysfunction of the left ventricle in 21% and systolic dysfunction of the right ventricle in 17%. The percentage of aortic regurgitation went up to 21%. The postoperative mortality rate at that time was high (14%). Surgical technique and pre- and postoperative care have improved dramatically since that era given the current mortality rate of 0%. It is unknown if these long-term complications will appear in our cohort. It would be interesting to evaluate late echocardiographic results in our patients. New long-term follow-up studies should be performed to see whether the long-term outcome has changed.

Our aim was to define risk factors for complications and major adverse events. Because complications and major adverse events are rare, multivariate analysis was not possible in our study. In order to actually define risk factors for complications and major adverse events future research with larger cohorts should be conducted.

### Limitations

The limitations of this study are primarily defined by the limitations of all retrospective chart studies. In a prospective study more information could be assessed and the length of follow-up could be more consistent. Secondary this was a single institutional experience so our findings may not be applicable to other centers.

## Conclusion

Contemporary results of surgical VSD closure are excellent with no mortality and low morbidity in this series. Although it is associated with increased ventilation time and a longer hospital stay low bodyweight at operation is not associated with an increased risk of complications or major adverse events in our series.
